# Do Metals Meddle with Puberty in Girls? Lead, Cadmium, and Altered Hormone Levels

**DOI:** 10.1289/ehp.118-a542b

**Published:** 2010-12

**Authors:** Kellyn S. Betts

**Affiliations:** **Kellyn S. Betts** has written about environmental contaminants, hazards, and technology for solving environmental problems for publications including *EHP* and *Environmental Science & Technology* for more than a dozen years

Lead and cadmium are both known reproductive toxicants. Now researchers have identified a link between relatively low levels of these metals and hormone markers of delayed onset of puberty in girls **[*EHP*** **118(12):1782–1787; Gollenberg et al.]**.

A team of scientists led by researchers at the National Institute of Child Health and Human Development used blood samples collected from girls aged 6–11 years as part of the nationally representative Third National Health and Nutrition Examination Survey, conducted by the Centers for Disease Control and Prevention (CDC) between 1988 and 1994. The team measured concentrations of two reproductive hormones—inhibin B and luteinizing hormone—that serve as markers of hypothalamic, pituitary, and gonadal functioning.

Associations with lead were estimated for luteinizing hormone in 671 girls and inhibin B in 655 girls. Most of the girls whose hormones were measured had blood lead levels below the CDC’s 10-μg/dL action level. The median blood lead level was 2.5 μg/dL, and less than 20% of the girls had blood lead levels exceeding 5 μg/dL. The median urinary cadmium concentration was 0.12 ng/mL (the authors considered levels over 0.27 ng/mL to be high). Non-Hispanic black girls had higher age-adjusted levels of both lead and cadmium than non-Hispanic whites or Mexican Americans.

The researchers found no significant associations with luteinizing hormone. However, girls aged 10 or 11 with blood lead levels of 5 μg/dL or higher were 75% less likely than girls with blood lead under 1 μg/dL to have levels of inhibin B greater than 35 pg/mL, a level typically deemed consistent with puberty by the limited research in this area. The researchers also found proportionately lower levels of inhibin B in girls who had relatively high levels of both cadmium and lead, compared with girls who had only high lead. Moreover, after adjusting for age, inhibin B levels were lowest for iron-deficient girls with blood lead levels of 1 μg/dL or higher, suggesting that lead may be particularly toxic for girls with iron deficiency.

The authors conclude that lead may suppress the production of hormones associated with puberty, especially in concert with cadmium. They stress that, on a national scale, changes in the timing of onset and/or progression of puberty can have considerable public health and social implications for both boys and girls. For instance, relatively late-maturing girls are at risk for diminished bone strength and fragility fractures later in life. The hormone alterations linked to lead and cadmium exposure in the study also could have other as-yet unknown effects.

